# Dimming the corona: studying SARS-coronavirus-2 at reduced biocontainment level using replicons and virus-like particles

**DOI:** 10.1128/mbio.03368-23

**Published:** 2024-11-12

**Authors:** Grace Neilsen, Asha Maria Mathew, Jose M. Castro, William M. McFadden, Xin Wen, Yee T. Ong, Philip R. Tedbury, Shuiyun Lan, Stefan G. Sarafianos

**Affiliations:** 1 Laboratory of Biochemical Pharmacology, Department of Pediatrics, Center for ViroScience and Cure, Emory University School of Medicine, Atlanta, Georgia, USA; 2 Children’s Healthcare of Atlanta, Atlanta, Georgia, USA; Albert Einstein College of Medicine, Bronx, New York, USA

**Keywords:** SARS-CoV-2, COVID-19, replicon, virus-like particles, coronavirus, drug discovery, vaccines

## Abstract

The coronavirus-induced disease 19 (COVID-19) pandemic, caused by severe acute respiratory syndrome coronavirus-2 (SARS-CoV-2) infections, has had a devastating impact on millions of lives globally, with severe mortality rates and catastrophic social implications. Developing tools for effective vaccine strategies and platforms is essential for controlling and preventing the recurrence of such pandemics. Moreover, molecular virology tools that facilitate the study of viral pathogens, impact of viral mutations, and interactions with various host proteins are essential. Viral replicon- and virus-like particle (VLP)-based systems are excellent examples of such tools. This review outlines the importance, advantages, and disadvantages of both the replicon- and VLP-based systems that have been developed for SARS-CoV-2 and have helped the scientific community in dimming the intensity of the COVID-19 pandemic.

## INTRODUCTION

Coronaviruses represent a significant threat to global health, causing infectious diseases that can range from innocuous to fatal. While some coronaviruses are endemic and cause minor cold-like symptoms (e.g., human coronavirus OC43) [reviewed in reference (1)], several recent spillover events from other animal carriers such as bats have introduced deadly strains into the human population, causing epidemics (2, 3) culminating with the recent coronavirus-induced disease 19 (COVID-19) pandemic (4–6). The 2003 severe acute respiratory syndrome coronavirus (SARS-CoV) epidemic and the 2012 Middle East respiratory syndrome (MERS) coronavirus epidemic both exhibited severe illness and high death rates but were geographically contained epidemics (7, 8). However, the more recent spread of SARS-CoV-2, the causative agent of the COVID-19 pandemic (4), has affected the world, causing almost 776 million confirmed cases and over 7 million deaths in the past 4 years (9). SARS-CoV-2 has also had an estimated cost of $8.1 to $15.8 trillion US dollars globally (10). The COVID-19 pandemic has supercharged the search for antiviral drugs [reviewed in references (11, 12)] and intensified the need to understand coronavirus biology as zoonotic transfers will likely continue with increased interactions between humans and previously remote animal populations (10, 13, 14). Further research into SARS-CoV-2 will better prepare the world for the next coronavirus epidemic.

SARS-CoV-2 primarily spreads through respiratory droplets and aerosols produced by infected individuals during activities such as coughing, sneezing, speaking, or breathing (15). Infection can result in a spectrum of symptoms, ranging from mild to severe, including fever, cough, shortness of breath, loss of taste or smell, and, in some cases, organ failure or death (16). The virus has evolved into several variants of concern (VOCs) throughout this pandemic, including the current Omicron strains (www.who.int). Vaccines (17–20) and therapeutic monoclonal antibodies [reviewed in reference (21)] have been developed. However, their efficacy has been challenged by the rapid evolution of new viral strains. Moreover, the use of vaccines in the later stages of the pandemic has significantly decreased (22, 23). Some antiviral drugs have been developed and used to treat COVID-19 as of 2024, including remdesivir (24), molnupiravir (25), paxlovid (nirmatrelvir with ritonavir) (26), and ensitrelvir (27). While these drugs have been employed with some success, antiviral resistance and the constant evolution of new strains and VOCs may eventually erode the effectiveness of these treatments. Accordingly, research continues into improved SARS-CoV-2 inhibitors, important virus-host interactions, and general coronavirus biology to develop information and tools that will be critical for the prevention of future coronavirus outbreaks.

A major technical hurdle for studying SARS-CoV-2 is the containment needed for experiments with a fully infectious virus. SARS-CoV-2 is labeled as a biosafety level 3 (BSL-3) agent, meaning it can only be used in BSL-3 laboratories by trained personnel (28). This severely limits the speed and breadth of research that can be conducted since not all research groups have access to BSL-3 facilities (29). However, several systems have been developed to study viruses in BSL-2 conditions, including replicon and virus-like particle (VLP) systems. This review focuses on the development and current research into various SARS-CoV-2 replicon and VLP systems and the advantages of each for drug development and biological studies.

## SARS-COV-2 LIFE CYCLE

**Fig 1:**
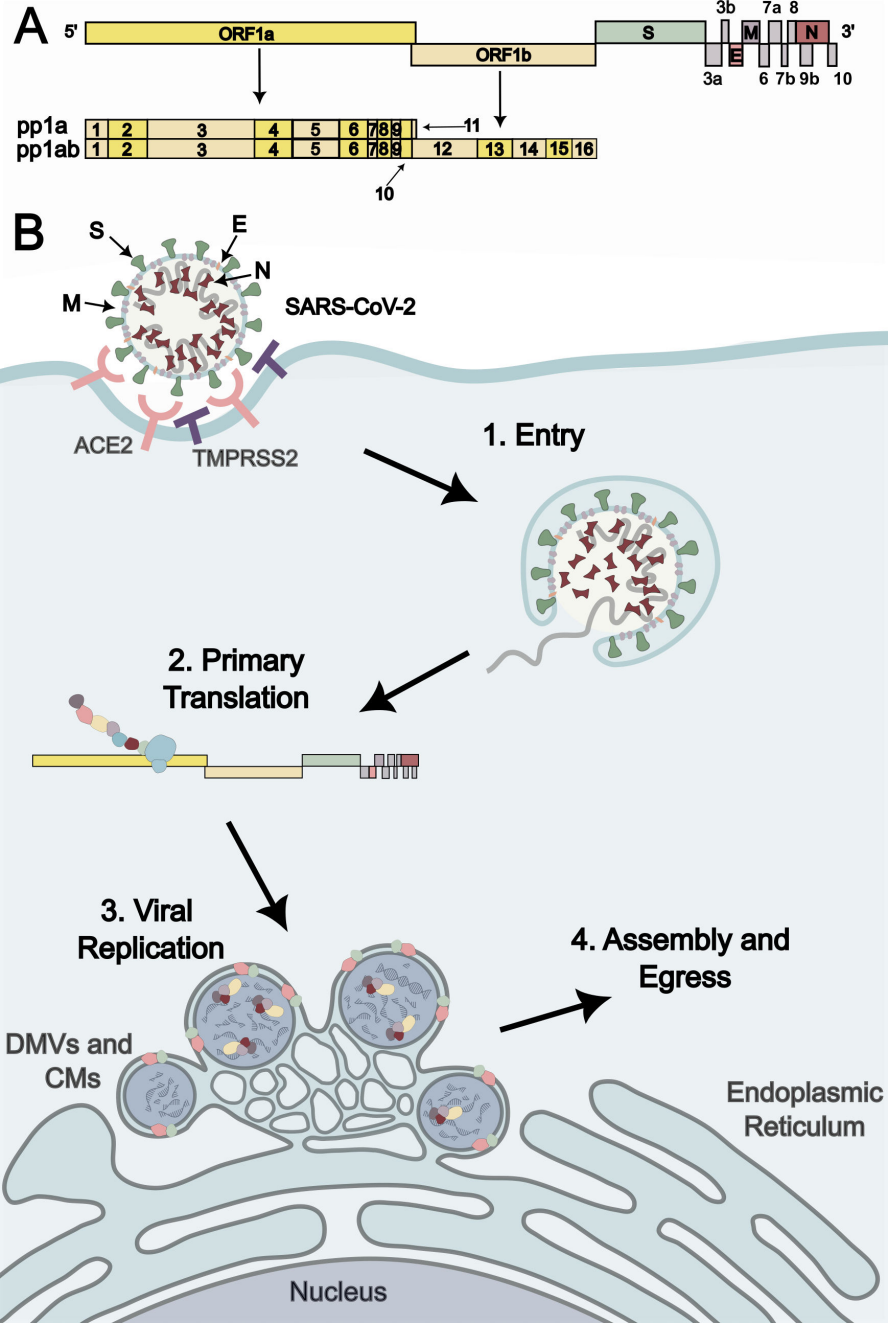
SARS-CoV-2 genome and early life cycle. (**A**) SARS-CoV-2 genome organization includes ORF 1a and 1b, which result in the polyproteins 1a and 1ab (pp1a and pp1ab) comprising 16 nsps; nsp1, disrupting host messenger RNA translation; nsp2, disrupting signal transduction; nsp3, multidomain protein involved in polyprotein processing [papain-like protease (PLpro)], double-membrane vesicle (DMV) formation, and immune evasion; nsp4, DMV formation; nsp5, main protease for polyprotein processing [3C-like protease (3CLpro)]; nsp6, DMV formation; nsp7/8, scaffold proteins for replication-transcription complex; nsp9, RNA-binding protein; nsp10, binder/scaffold of nsp14; nsp12, RNA-dependent RNA polymerase and nucleotidyltransferase (NiRAN) activities; nsp13, helicase; nsp14, exoribonuclease activity (proofreading); nsp15, endonuclease (EndoU) degrades dsRNA; nsp16, 5′ cap formation. Other ORFs code for the structural proteins (S, E, M, and N) and accessory proteins (shown as gray boxes, ORF3a, ORF3b, ORF6, ORF7a, ORF7b, ORF8, ORF9b, and ORF10). (**B**) SARS-CoV-2 life cycle. After a SARS-CoV-2 virion enters the cell, host ribosomes translate the released positive-sense RNA genome. Some viral proteins are embedded in the endoplasmic reticulum (ER) where they contort the ER membrane into DMVs and other convoluted membrane (CM) structures to hide the replication-transcription complex, and replication intermediates, from the innate immune system as it synthesizes viral RNA.

SARS-CoV-2 is from the order Nidovirales family Coronaviridae designated a β-coronavirus in the Baltimore group IV capable of infecting mammals, including humans (30, 31). The enveloped virion’s diameter ranges between 60 and 140 nm and is covered by approximately 24 prefusion spike glycoprotein trimers giving the characteristic crown or “corona” silhouette (32, 33). SARS-CoV-2 is an enveloped, positive-sense, single-stranded RNA virus. The genomic RNA (gRNA) spans 29,903 nucleotides [in the Wuhan-Hu-1 strain (34)] with a 5′ cap structure and a 3′ poly-A tail (35). The genome encompasses 14 open reading frames (ORFs) that encode 29 proteins. The initial two-thirds of the genome code for the 16 non-structural proteins (nsps) in the form of ORF1a and ORF1b polyproteins, while the final third codes for the four structural proteins—spike (S), envelope (E), membrane (M), and nucleocapsid (N)—along with nine accessory factors (35, 36) (Fig. 1A). Understanding the SARS-CoV-2 replication cycle and the proteins involved is crucial in developing effective replicon and VLP systems.

### Entry

SARS-CoV-2 enters the host cell membrane *via* the interaction between the viral S protein and host angiotensin-converting enzyme 2 receptor (37, 38). Following receptor binding, S undergoes cleavage facilitated by either the transmembrane protease serine 2 (TMPRSS2) or endosomal cathepsin L, leading to the fusion of the viral and cellular membranes, thus releasing the RNA genome into the host cytoplasm (39–41).

### Primary translation

The newly released gRNA allows for the immediate translation of polyprotein 1a and 1ab (pp1a and pp1ab). Pp1a is encoded by ORF1a, and pp1ab is the result of a programmed −1 ribosomal frameshift resulting in the readthrough of the stop codon at the end of ORF1a into ORF1b (Fig. 1A) (42, 43). The polyproteins are further processed by non-structural protein 3 (nsp3) (a papain-like protease; PLpro) (44–46) and nsp5 [also known as 3C-like protease (3CLpro) or main protease (Mpro)] present within the polyproteins (47, 48). These processing events give rise to 16 nsps that play important roles in the viral replication cycle (Fig. 1).

### Viral replication

To concentrate the replication machinery and evade innate immune sensors, the nsp3, nsp4, and nsp6 together rearrange the endoplasmic reticulum (ER) membrane to form double-membrane vesicles (DMVs), the site of viral replication (49–52). Nsps 7–16 are responsible for the various steps in the amplification of the viral genome within the DMVs. The replication-transcription complex (RTC) comprises the RNA-dependent RNA polymerase nsp12 and the scaffold proteins nsp7 and nsp8 (53). With the help of a helicase, nsp13, the RTC is able to transcribe negative-sense copies of the viral gRNA (54). The newly replicated RNA is proofread by the exoribonuclease, nsp14, which together with the scaffold protein nsp10 sits on the back of the RTC (55–58). In addition to the continuous replication of the negative-sense RNA template by nsp12, the polymerase generates 3′ and 5′ subgenomic RNAs (sgRNAs) *via* discontinuous transcription (59–62). This process produces negative-strand sgRNAs that are re-transcribed by the RTC to generate positive-sense sgRNAs needed for the translation of the structural and accessory proteins (59, 60). The nascent transcripts and replicated gRNA contain a 5′ cap, orchestrated by a series of reactions involving nsps 12–14 and 16 and a polyadenylated tail to evade the cellular immune response, increase RNA stability, and enable efficient translation by host ribosomes (63–73). Nsp1 aids sgRNA translation by binding ribosomes to inhibit host messenger RNA (mRNA) translation while allowing viral sgRNA translation (74). To abate immune response to the production of sgRNA, nsp15 endoribonuclease (EndoU) reduces viral dsRNA by degradation attenuating interferon signaling (75–77). Nsp2 has been implicated in regulating infection by disrupting signal transduction in infected cells and intersecting stress-derived cellular responses preventing apoptosis (78, 79).

### Assembly and egress

From the positive-sense sgRNAs, the S, E, and M proteins are translated at the ER and then trafficked through the endoplasmic reticulum-Golgi intermediate compartment (ERGIC) for further processing (80). Meanwhile, N is translated in the cytosol and then binds to the viral RNA genome, forming the ribonucleoprotein (RNP) complex. The RNP complex and the S, E, and M proteins assemble into immature virions at the ERGIC (80). The virions then bud into the ERGIC lumen and undergo further maturation to acquire their final morphology. Finally, mature virions are transported in lysosomes to the plasma membrane and are released from host cells through exocytosis (81, 82).

## WHAT ARE REPLICONS?

Replicons are self-replicating subgenomic viral systems that carry essential genes for viral genome replication but typically lack structural genes required for viral structure and assembly. In these systems, the coding sequences for structural proteins are typically replaced with reporters or selectable markers, such as luciferase or green fluorescent protein (GFP). This replacement enables convenient detection and quantification of viral replication while preserving the proteins and nucleic acid sequences necessary for genome replication. Replicons are used for studying viral replication, conducting antiviral screening, developing VLPs through encapsidation of structural proteins toward the development of vaccines, as well as identifying and characterizing mutations that confer resistance to antiviral drugs without the risk of gain-of-function (83).

Early work on hepatitis C virus (HCV) replicon systems played a crucial role in early HCV studies. At the time, it was difficult to grow a fully infectious virus; HCV replicon studies greatly advanced our understanding of the HCV replication cycle and advanced drug discovery efforts through high-throughput screening of chemical libraries that led to curative therapies for hepatitis C (84–89). These replicons, which replicate in human hepatoma Huh7 cells, contain the viral 5′ and 3′ untranslated regions (UTRs) and utilize internal ribosome entry sites (IRES) for translation. The first ORF encodes the selectable neomycin phosphotransferase gene, while the second ORF translates the HCV non-structural proteins necessary for viral replication (84, 85). HCV replicons have also been reported to acquire adaptive mutations with enhanced replication phenotype (86).

Over the past decades, RNA replicons have been generated for various positive-sense single-stranded RNA (+ssRNA) viruses (90–108), including, but not limited to, dengue virus (109, 110), Zika virus (111, 112), SARS-CoV (113), and SARS-CoV-2 (114–136). These viruses possess a genome that functions as messenger RNA, enabling direct translation by the host cell ribosomes.

### SARS-CoV-2 replicon systems

**Fig 2:**
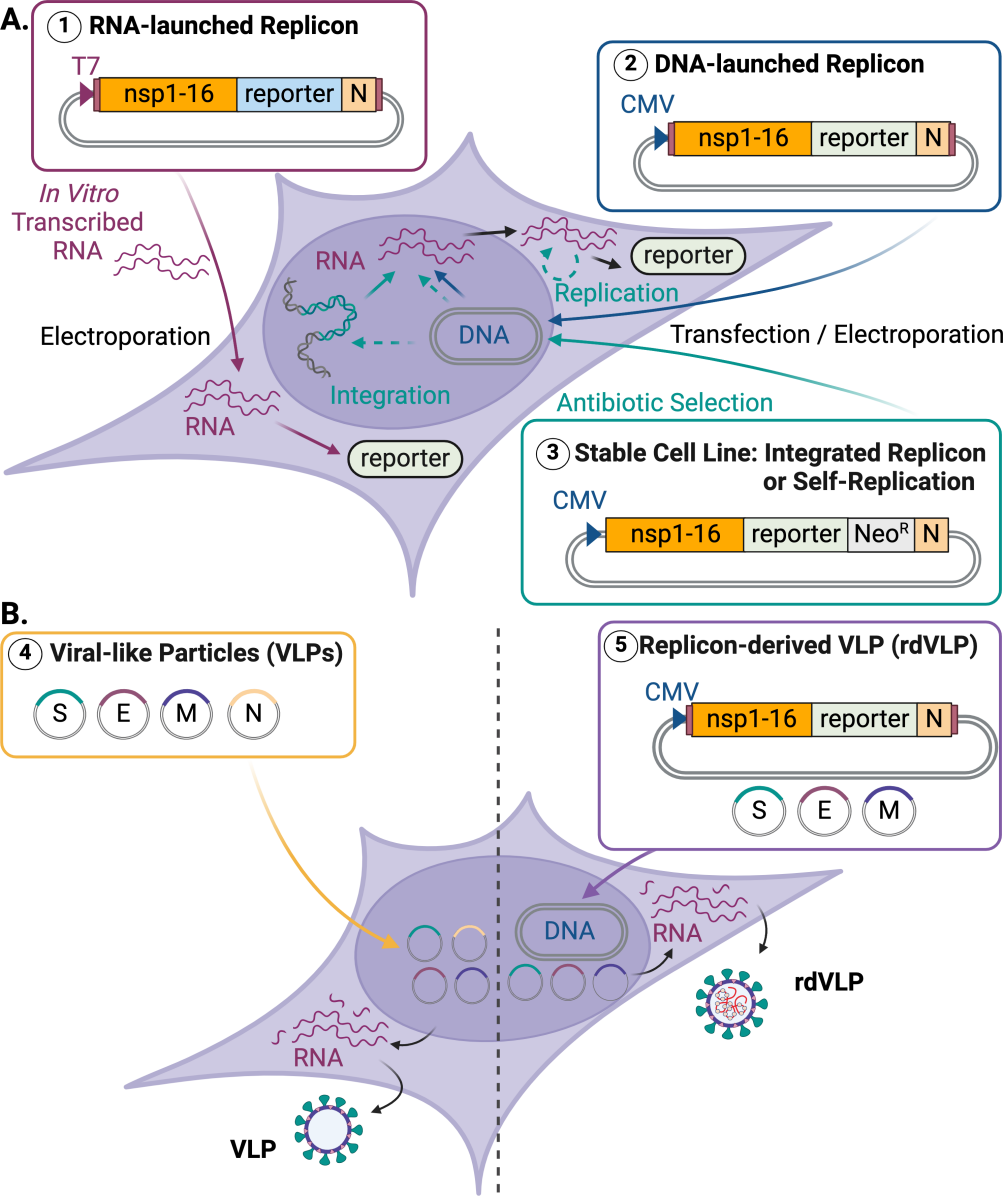
Schematic of SARS-CoV-2 replicon systems and VLP reporter strategies. (**A**) Replicon systems generate a translated reporter signal encoded by electroporated RNA transcribed *in vitro* (1) or are encoded by mRNA transcribed by cellular RNA polymerases from a DNA template, e.g., transfected or electroporated DNA plasmids (2) or stable cell lines that either persistently harbor SARS-CoV-2 replicon by containing replicon-coding DNA integrated into their genome or are based on self-replicating RNA expressing a selectable reporter (3). (**B**) Examples of VLP (4) and replicon-derived VLP (5) systems. Expressing the structural proteins (S, E, M, and N) in producer cells results in the production of empty VLPs (4). VLPs can enter target cells through receptors, but they cannot undergo replication inside them. Co-transfection of a replicon with plasmids expressing these structural proteins results in rdVLPs (5). rdVLPs can package the replicon and thus can both enter target cells and undergo one round of infection. In similar approaches, the N protein can be omitted from the replicon genome or S, E, and M (but not all three) may be included in the replicon sequence. Promoters other than CMV (cytomegalovirus) may also be used. Figure created with BioRender.

During the first SARS-CoV epidemic, replicon systems were developed to help study SARS-CoV (113). These early efforts helped with the development of a wide variety of SARS-CoV-2 replicon systems during the subsequent COVID-19 pandemic (Table 1). The general genomic composition of SARS-CoV-2 replicons typically includes (i) ORF1a/1b comprising the 16 nsps (1–16, listed in Fig. 1), (ii) reporter genes, (iii) selection markers, (iv) N (to minimize innate immune response), and (v) 5′ and 3′ untranslated regions. However, replicons vary in the method used for viral RNA production. Most RNA-launched replicons involve T7 promoter-driven *in vitro* transcription and electroporation into permissive cells (Fig. 2A-1) (115, 118–122, 126, 128, 130–134). Other examples are DNA-launched replicons, with many utilizing the human cytomegalovirus (CMV) immediate-early promoter to transcribe RNA from the transfected plasmid DNA into cells (Fig. 2A-2) (114, 116, 117, 120, 123–125, 127, 129, 131). Several coronavirus replicons have been constructed with bacterial artificial chromosome (BAC)-derived plasmids (85, 134, 135). BACs are circular, self-replicating vectors that carry large DNA insertions and typically maintain only one or two copies per *Escherichia coli* cell, which helps stabilize these insertions and reduce insert-associated cytotoxicity (132, 133).

**TABLE 1:** List of SARS-CoV-2 replicons

No.	Information	RNA launched	DNA launched	Stable cell line	ORF 2–8 deletion	Reporter or selection marker	Reference
1	T7 promoter *in vitro*-transcribed RNA	**+**			**+**	*Renilla* luciferase and neomycin phosphotransferase	(130)
2	T7 promoter *in vitro*-transcribed RNA	**+**			**+**	Firefly luciferase and GFP	(115)
3	T7 promoter *in vitro*-transcribed RNA with N deletion	**+**			**+**	GFP	(118)
4	T7 promoter *in vitro*-transcribed RNA	**+**			**+**	HiBiT tag	(119)
5	T7 promoter *in vitro*-transcribed RNA	**+**				*Gaussia* luciferase or mNeonGreen	(126)
6	CMV promoter-driven DNA		**+**		**+**	TurboGFP-blasticidin or firefly luciferase	(128)
7	T7 promoter *in vitro*-transcribed RNA	**+**			**+**	*Renilla* luciferase	(132)
8	T7 promoter *in vitro*-transcribed RNA with ORF3-E deletion	**+**				mNeonGreen	(133)
9	T7 promoter *in vitro*-transcribed RNA	**+**			**+**	*Gaussia* luciferase	(134)
10	CMV promoter-driven DNA		**+**		**+**		(117)
11	CMV promoter and IRES, multiple plasmids		**+**		**+**	GFP and luciferase	(123)
12	CMV promoter-driven DNA		**+**		**+**	Nano luciferase	(125)
13	T7 promoter *in vitro*-transcribed RNA or CMV promoter-driven DNA	**+**	**+**	**+** (BHK-21 and HEK293 cells)	**+**	GFP or mNeonGreen with Neomycin phosphotransferase II and nano luciferase	(120)
14	T7 promoter, SARS-CoV-2-Rep-NanoLuc-Neo	**+**		**+** (BHK-21 cells)	**+**	Nano luciferase and neomycin phosphotransferase II	(121)
15	CMV promoter-driven DNA		**+**		**+**	eGFP	(114)
16	HIV long terminal repeat/T7 dual-promoter-driven DNA/RNA	**+**	**+**		**+**	Firefly luciferase and GFP	(122)
17	CMV promoter-driven DNA		**+**		**+**	*Gaussia* luciferase and GFP	(124)
18	CMV promoter-driven DNA		**+**	**+** (VeroE6/Rep3 cells)	**+**	*Renilla* luciferase	(127)
19	T7 promoter-driven *in vitro*-transcribed RNA or CMV promoter-driven DNA	**+**	**+**		**+**	Nano luciferase	(131)
20	CMV promoter-driven DNA		**+**		**+**	mNeonGreen	(129)
21	CMV promoter-driven DNA		**+**		**+**	mNeonGreen	(116)
22	T7 promoter-driven *in vitro*-transcribed RNA	**+**			**+**	HiBiT tag	(135)
23	CMV promoter-driven DNA		**+**		**+**	Nano luciferase	(136)

#### RNA-launched SARS-CoV-2 replicons

T7 promoter-based SARS-CoV-2 replicons require *in vitro* transcription and RNA extraction prior to electroporation into permissive cells. Several studies have utilized T7 promoter-driven systems to construct SARS-CoV-2 replicons with reporter genes. The first T7 promoter-based SARS-CoV-2 replicon was designed through the *in vitro* ligation of fragments carried in seven separate plasmids, involving the replacement of genes S, E, M, and accessory genes with a gene cassette encoding *Renilla* luciferase, an FMDV 2A self-cleaving sequence, and neomycin phosphotransferase (Neo). This replicon RNA was assessed in Huh7 and BHK-21 cells (130). Furthermore, a HiBiT tag reporter integrated into the C-terminus of the N protein was employed in an *in vitro*-ligated replicon DNA with a T7 promoter (119) or pCC2Fos vector (135). The *in vitro* ligation approach offers the advantages of rapidly manipulating individual fragments for cloning and mutagenesis. However, it may result in a low yield of full-length replicon products. Ricardo-Lax and colleagues (126) reported a system where yeast transformation-associated recombination was used to assemble T7 promoter-based replicons with *Gaussia* luciferase and mNeonGreen reporters, and phi29 DNA polymerase was employed to amplify replicon plasmids. *In vitro*-transcribed replicon RNA has been characterized in various cell lines, including BHK-21, Huh7.5, Vero E6, Caco-2, Calu-3, NHBE, NHLF, and A549 (126).

Other T7 promoter-based SARS-CoV-2 replicons use BAC plasmids, with a T7 minimal promoter upstream of the 5′ UTR and nsp1-16 followed by luciferase and GFP reporters, alongside a cassette comprising a 26-nucleotide poly(A) sequence, a hepatitis D virus ribozyme, and a T7 terminator at the 3′ end to ensure precise viral genome termination (115). Similar T7 promoter-driven *in vitro*-transcribed replicon systems have been reported with single *Renilla* luciferase (132), *Gaussia* luciferase (134), or nano luciferase-blasticidin reporter (131). Incorporating GFP, neomycin phosphotransferase II (NPTII), and nano luciferase reporters, our group also developed a replicon using T7 promoter-driven *in vitro*-transcribed RNA, which was characterized in BHK-21 and HEK293T cells (120).

#### DNA-launched SARS-CoV-2 replicons

Other SARS-CoV-2 replicons using transcriptional promoters were also developed, including plasmid DNA-based replicons constructed with the CMV promoter and GFP and luciferase reporters, expressing different nsp proteins (123). Although replicon activity depends on the efficiency of multiple plasmid transfections, this strategy is convenient for manipulating different genes for functional analysis. A replicon assembled by overlap extension PCR with 26 gene fragments and transformation-associated recombination in yeast was reported (122). This whole replicon genome was placed under the control of a CMV promoter and engineered with turboGFP, blasticidin deaminase, and firefly luciferase genes (128). Our group also developed replicons using CMV promoter-driven DNA, incorporating GFP or mNeonGreen, NPTII, and nano luciferase reporter (120). These replicons were characterized in multiple cell lines, including HEK293T, BHK-21, CHO-K1, A549, Huh7, Vero E6, Caco-2, and Calu-3 (120). Other CMV promoter-driven replicons with variable reporter and selection genes have also been published, including mNeonGreen- or luciferase-puromycin (117), nano luciferase-neomycin phosphotransferase II (125), nano luciferase-blasticidin (131), eGFP (114), or mNeonGreen (116, 129). An elevated level of replicon Nluc reporter signal was reported through inhibiting RNA splicing with a universal eukaryotic host splicing inhibitor, isoginkgetin (136). Additionally, the He group constructed a SARS-CoV-2 replicon with firefly luciferase and GFP reporter driven by HIV long terminal repeat/T7 dual-promoter, where NSP2-16 was translated through an IRES (122). While T7 promoter-based replicons better resemble the direct delivery of viral RNA, CMV promoter-driven SARS-CoV-2 replicons are notable for their ease of preservation, manipulation, and application.

#### Stable cell line-harbored SARS-CoV-2 replicons

Three groups have established stable cell lines expressing SARS-CoV-2 replicon (Fig. 2A-3). We have successfully generated SARS-CoV-2 replicon-expressing stable cell lines by employing CMV promoter-driven BAC DNA, incorporating orthogonal GFP and nano luciferase reporter genes along with neomycin phosphotransferase II for selection (120). This work resulted in stable human, HEK293, and BHK-21 cell lines (120). The Tanaka group (127) generated non-human stable cell lines (VeroE6/Rep3) expressing SARS-CoV-2 replicon. Finally, the Liu group (121) reported stable cell lines with a T7 promoter-based replicon (SARS-CoV-2-Rep-NanoLuc-Neo) by replacing the S gene with a nano luciferase reporter gene and an NPTII selection gene. In this system, to facilitate stable cell line selection, they introduced nsp1 K164A/H165A mutations to reduce cellular toxicity.

### Advantages and limitations of SARS-CoV-2 replicon systems

The SARS-CoV-2 replicon systems enable researchers to study SARS-CoV-2 biology and screen for antiviral compounds at a less stringent biosafety level than that required for live virus (e.g., BSL-2 vs BSL-3). Replicon systems are excellent tools for quick and efficient high-throughput screening to identify potential antiviral drugs. They can also be used for studying specific aspects of viral replication, transcription, and protein expression without the complexity of a fully infectious virus, making it easier to dissect the roles of individual viral proteins and RNA elements. The replicon system can be engineered to include reporter genes (e.g., luciferase or GFP), which simplifies the monitoring of viral replication and gene expression and allows the real-time tracking of viral activity and evaluation of potential inhibitors. These systems can also be easily engineered to study the effect of mutations or modifications on viral replication. Interestingly, alphavirus-derived replicons have been used as a platform to deliver SARS-CoV-2 S as a vaccine (137–142).

SARS-CoV-2 replicon systems are powerful tools that can accelerate research and reduce safety risks, but they come with limitations. As they lack the S, E, and M structural genes, they do not fully recapitulate all aspects of the viral replication cycle and interactions with host cells. Moreover, since they are typically delivered to target cells *via* transfection, they are only used in the context of suitable cell lines and not primary cells. Since they do not produce infectious viruses, the replicon systems can only provide limited information on how the virus spreads, causes host pathology, or elicits an immune response. Also, standard replicon systems cannot be used to evaluate compounds acting on receptor binding, virus entry, encapsidation, and virus release. However, more advanced replicon systems that can address these steps are described below [replicon-derived virus-like particles (rdVLPs)].

## WHAT ARE VIRUS-LIKE PARTICLES?

VLPs have been used to study diverse viruses over the past two to three decades. They are assemblies of virus structural proteins that mimic the morphology of a native virus, except that they lack critical elements such as the viral genetic material. VLPs may present viral spikes and other surface components in a repetitive array (143, 144). VLPs typically do not contain genetic material, so they are incapable of replicating and producing infectious viruses. When used for vaccine studies, VLPs elicit an immune response akin to a challenge with the authentic virus (145). VLP vaccines are more immunogenic than subunit vaccines, and they can induce mucosal and systemic immunity (146–148). Thus, the VLP platform has enormous potential for use as a viral vaccine strategy (145–148).

VLPs further serve as model systems to study the formation and assembly of macromolecular complexes and viral entry routes in different viruses. Frequently, viral structural proteins involved in capsid or envelope formation self-assemble into VLPs when co-expressed in cells even without viral components required for replication, such as non-structural proteins or some structural proteins (Fig. 2B-4) (144, 145). This allows VLPs to be used for studying the impact of mutations in the structural proteins on viral assembly, egress, and entry.

Depending on their structural composition, VLPs may be enveloped or nonenveloped and composed of one protein (homologous), several proteins from the same virus (heterologous), or several proteins from different viruses (chimeric), according to their viral components (146, 149–151). Many experimental procedures have been described that may help answer questions concerning the requirements for VLP formation (145). Understanding the assembly process of VLPs is critical to defining the effectiveness of such particles for the presentation of their own or foreign epitopes as carriers for transiently expressed proteins as a means of vaccine production (146, 148, 150). VLPs have been proposed in gene delivery, drug delivery, and gene editing technologies (152–156). Bioinformatics tools can also be used to improve and rationalize the design of new and pre-existing VLPs to achieve the best immunogenic performance (157).

### VLPs as vaccine candidates

Several VLP-based vaccines are licensed and are available in the market for Hepatitis B (Engerix-B from GlaxoSmithKline, Recombivax HB from Merck & Co.), Hepatitis E (Hecolin from Xiamen Innovax Biotech Co.), human papillomavirus (Cervarix from GlaxoSmithKline, Gardasil 9 from Merck & Co.), and against malaria (Mosquirix from GlaxoSmithKline) (153, 154, 158, 159).

Because of the scalability and success rate of VLP vaccines in industrial settings, efforts are being made to develop viable VLP-based vaccines against SARS-CoV-2. Such vaccines would provide a versatile and easily adaptable platform. Although mRNA-based vaccines were developed rapidly in response to the acute phase of the pandemic, a less expensive, adaptable vaccine ideal for studying variants and different mutations and combinations of mutations that mimic the native SARS-CoV-2 virion has been considered (160–162). A VLP-based vaccine platform may address these potential issues as they mimic the native viral structure and have the potential to elicit a high immune response.

### SARS-CoV-2 VLPs

Different host expression systems (bacterial, insect, plant, yeast, or mammalian) have been used to produce SARS-CoV-2 VLPs by co-expressing S, E, and M proteins (Fig. 2B-4). One of the first SARS-CoV-2 VLP studies was published by Xu et al. (156) in 2020, producing the VLPs in mammalian cells and establishing that E and M proteins are critical for VLP formation. Since 2020, a variety of systems have been utilized to develop SARS-CoV-2 VLPs, including a report on synthetic SARS-CoV-2 VLPs (163). Table 2 lists information on the different VLPs produced against SARS-CoV-2.

**TABLE 2:** SARS-CoV-2 virus-like particles

No.	Information	Expression system				Reference
		Bacterial	Insect	Mammalian	Other	
1	Co-expression of S, E, and M		**+**			(164)
2	Co-expression of M and E proteins (bicistronic), N protein, and HA-tagged S protein (both monocistronic)		**+**			(165)
3	Co-expression of S, E, and M			**+**		(166)
4	Co-expression of M, E, and S		**+**			(167)
5	Co-expression of M, N, E, and S			**+**		(168)
6	Co-expression of M, N, E, and S			**+**		(156)
7	Co-expression of M, N, E, and S			**+**		(169)
8	Co-expression of S, E, and M			**+**		(170)
9	Co-expression of N, E, and M				Plant	(171)
10	Co-expression of S, E, and M				Plant	(172)
11	Co-expression of S, E, and M		**+**			(173)
12	Co-expression of M, N, E, S, and a plasmid encoding luciferase mRNA linked to a SARS-CoV-2 packaging signal			**+**		(161)
13	Co-expression of M, N, E, and S			**+**		(174)
14	Expression of SARS-CoV-2 spike fused with an inﬂuenza A transmembrane and the cytoplasmic tail of HA and the M1 protein			**+**		(175)
15	Chimeric VLP of SARS-CoV-2 spike protein and H5N1 M1		**+**			(176)
16	Expression of M/N/E, or S or co-expression of their combinations			**+**		(177)
17	Co-expression of M, N, E, and S and HexaProline S			**+**		(178)
18	S protein fused with the TMCTD of VSV-G; S ectodomain fused with VSVS-G TM-CTD expressed on MLV gag eVLPs			**+**		(179)
19	Expression of prefusion S protein				Plant	(180)
20	HIV-1 Gag-based SARS-CoV-2 spike VLPs			**+**		(181)
21	Co-expression of S, E, and M				Plant	(182)
22	*Acinetobacter* phage 205 cVLP mixed with SARS-CoV-2 Spike RBD antigen	**+**				(183)
23	Plasmonic Au cores and S1-spike protein coronas				Synthetic	(163)
24	S, E, and M, with HCV core signal peptide between the S, E, and M genes			**+**		(184)
25	Co-expression of S, M, and E			**+**		(185)
26	Spike glycoproteins reconstituted into liposome formulations				Synthetic	(186)
27	Co-expression of S, M, or E with S-HA fusion protein and influenza M1		**+**			(187)
28	Recombinant SARS-CoV-2 S proteins and tail peptides, including constructs with enhanced mimicry of the coatomer-binding motif			**+**		(188)
29	Rabies glycoprotein containing S ectodomain			**+**		(189)
30	Different combinations of S/M/E and N proteins			**+**		(190)
31	Recombinant core antigen of HBV genotype G (HBc/G) and immunogenic sequences of S and N	**+**				(191)
32	ALVAC vector-based vaccine expressing the SARS-CoV-2 S, E, and M proteins			**+**		(192)
33	Chimeric SARS-CoV-2 S proteins bearing the cytoplasmic tail of either HIV-1 or SIV facilitating the formation of Gag-based VLPs			**+**		(193)
34	Newcastle disease virus-like particles displaying the prefusion-stabilized SARS-CoV-2 spike ectodomain (S2P)			**+**		(194)
35	RBM domain (aa437–508) of S is added to the C terminus of AP205 dimer	**+**				(195)
36	S or S-2P or S-6P displayed on MS2-SA VLPs	**+**				(196)
37	S or mini-spike expressing rhabdoviruses			**+**		(197)
38	Co-expression of S, E, and M				Yeast	(198)
39	Full length of spike (S) glycoprotein (S full), S1, or S2 together with the inﬂuenza matrix protein 1 (M1) as a core protein		**+**			(199)
40	Prefusion S combined with an adjuvant [Adjuvant System 03 (AS03)]				Plant	(200)
41	Trimeric full-length SARS-CoV-2 spike glycoproteins and Matrix-M1 adjuvant		**+**			(201)
42	Pseudotyping of MLV particles with different forms of S			**+**		(202)
43	Expression of full-length S				Plant	(203)
44	HBV core particles (HBc) were used as a platform for exposing SARS-CoV-2 epitopes in the major immunodominant region of HBc				Plant	(157)
45	A split-protein Tag/Catcher system was used to conjugate and display RBD antigen on the protein surface of preassembled bacteriophage AP205 cVLPs	**+**				(204)
46	Spike (RBD) on a synthetic virus-like particle platform, SpyCatcher003-mi3, using SpyTag/SpyCatcher technology	**+**				(205)
47	Cucumber mosaic virus VLPs containing the RBM from SARS-CoV-2 spike protein	**+**				(206)
48	Co-expression of codon-optimized S, E, and M		**+**			(207)

Multiple SARS-CoV-2 VLP candidates are currently at various stages of clinical trials for SARS-CoV-2 vaccination. Recently, the NVX-CoV2372 (Novavax) received FDA approval for emergency use and is the only commercially available VLP-based vaccine against SARS-CoV-2 (201, 208). These VLPs are produced by recombinant expression of full-length S protein in a baculovirus expression vector system, mixed with the saponin-based M adjuvant to create a ready-to-use vaccine. CoVLP, marketed under the brand name Covifenz, was a COVID-19 vaccine developed by Medicago in Canada in collaboration with GlaxoSmithKline using the Australian plant *Nicotiana benthamiana*. In February 2022, Health Canada approved CoVLP for use in preventing COVID-19 in adults aged 18–64 (180). The approval noted a 71% efficacy rate after two doses against COVID-19 symptoms and 100% efficacy against severe cases. However, in February 2023, Mitsubishi, the owner of both the product and Medicago Inc., terminated the company and its program. VBI-2902 is a VLP-based vaccine developed by VBI Vaccines that includes a modified, optimized, prefusion form of the SARS-CoV-2 spike antigen (179). The vaccine is in various stages of clinical trials, focusing on its efficacy and safety.

### Advantages and limitations of SARS-CoV-2 VLPs

SARS-CoV-2 VLPs have several unique advantages over other vaccine candidates. Some VLPs mimic the native SARS-CoV-2 virus but lack the genetic material to replicate and spread in cells, so they can replace live viruses in different assays (167). The time required to produce VLPs may be short, making them a good candidate for studying variants and different mutations (161, 162). For handling the SARS-CoV-2 infectious virus, BSL3 containment is required. When VLPs of SARS-CoV-2 are designed to be non-infectious, they can be handled in lower biosafety containment and replace the infectious virus in neutralization studies (177). SARS-CoV-2 VLPs are a good model system to study the morphology and structure of the virus and compare structural differences among different subtypes, in the context of an almost native structural environment that is relatively easy to engineer and prepare at quantities required for structural studies (149, 156, 162, 163). VLPs are also versatile and can be used to study viral entry, viral budding, and post-translational modifications (169, 182). However, they cannot be used to study aspects of the viral replication or antivirals that target replication, nor do they fully recapitulate the native conditions of infection in the target cell.

### Replicon-derived virus-like particles and related systems

To combine the utility of the replicon and VLP systems and enable studies on the complete replication cycle, our lab has recently developed a replicon-derived VLP system by co-transfection of the replicon plasmid with plasmids expressing the missing structural proteins (M, E, and S proteins) provided in *trans* (120). The resulting rdVLPs have authentic morphology, similar to other VLPs, but are also genetically capable of a single round of infection and extensive production of viral proteins, as they contain the replicon plasmid (Fig. 2B-5). Such a system can interrogate all aspects of the virus life cycle and is a powerful tool for testing antivirals and other compounds that may inhibit any step of viral replication. It may also be used to study the role of host factors in viral replication and is an attractive candidate for vaccine studies. The modular basis of this system enables facile molecular engineering studies where the effect of mutations that appear in variants of concern or drug-resistant strains can be examined in an expedited manner under BSL2 biocontainment. The rdVLP system is ideal for developing new antivirals targeting SARS-CoV-2, as it is sensitive, quantitative, and scalable to high-throughput workflows (120).

Other groups have developed similar strategies using both DNA- and RNA-based replicons to generate replication-competent VLP systems. Ricardo-Lax and colleagues reported that SARS-CoV-2 replicons trans-complemented with the spike protein could generate single-cycle virions called replicon-derived particles; these carried a mutated form of nsp1 and showed more efficient *trans*-complementation than the respective wild-type systems (126). A T7 promoter-driven *in vitro*-transcribed RNA replicon with mNeonGreen reporter and ORF3-E deletion was used to generate single-round virions in a Vero E6 cell line expressing the ORF3 and E proteins under a doxycycline-inducible promoter (133). A SARS-CoV-2 replicon with a tandem *Gaussia* luciferase and neon GFP dual reporter was also utilized to create a single-cycle infectious SARS-CoV-2 virus replicon particle system (124).

## FUTURE PERSPECTIVES

Replicons and VLPs should continue to be essential tools for developing therapeutics and vaccines for SARS-CoV-2 as well as future viral threats. The flexibility of constructing chimeric VLPs by expressing various viral proteins from the same or different virus families makes them formidable tools for developing vaccines capable of targeting multiple viruses simultaneously. Additionally, maintaining a library of different replicon and VLP systems, including rdVLPs, that can be quickly modified using straightforward molecular biology techniques is crucial for effectively combating emerging viral threats. For viruses categorized as high risk, the advancement of serological and immunological assays using VLPs could dramatically reduce the reliance on BSL-3 and BSL-4 laboratories, broadening the range of researchers able to contribute to these critical health challenges. Furthermore, scaling up VLP production for vaccine development is a crucial challenge that must be addressed. There is a need for increased research efforts to establish efficient protocols and novel methods for the mass production and purification of VLPs, particularly for industrial-scale production. Addressing these challenges will enhance our preparedness and response capabilities for future pandemic crises.
